# Hepatitis C Virus Potentially Transmitted by Opioid Drug Diversion from a Nurse — Washington, August 2017–March 2018

**DOI:** 10.15585/mmwr.mm6816a3

**Published:** 2019-04-26

**Authors:** Henry N. Njuguna, Denise Stinson, Patricia Montgomery, Nigel Turner, Marisa D’Angeli, Jason Carr, Sara Podczervinski, Cathy Wasserman, Sumathi Ramachandran, Todd Lucas, Danae Bixler, Kiran Perkins, Isaac Benowitz, Anne Moorman

**Affiliations:** ^1^Epidemic Intelligence Service, CDC; ^2^Washington State Department of Health; ^3^Tacoma-Pierce County Health Department, Tacoma, Washington; ^4^Division of Viral Hepatitis, National Center for HIV/AIDS, Viral Hepatitis, STD, and TB Prevention, CDC; ^5^Division of Healthcare Quality Promotion, National Center for Emerging and Zoonotic Infectious Diseases, CDC.

During January 22–March 23, 2018, a local health department in Washington was notified of two patients who received a diagnosis of acute hepatitis C virus (HCV) infection. Neither patient had behavioral risk factors associated with HCV acquisition; however, both had received injectable narcotic (opioid) drugs from the same nurse during separate visits to an emergency department (ED) at a local hospital on December 6 and December 16, 2017. Investigation revealed that the nurse had accessed the automated drug dispensing system at a higher frequency than had other staff members, admitted diverting* patients’ injectable narcotic and antihistamine drugs for personal use, and tested positive for HCV antibodies (anti-HCV) on March 19, 2018, but did not have quantifiable HCV RNA. Specimens from both patients were sent to CDC for genetic testing, and HCV viral variants analysis found a significant level of genetically similar HCV variants in both patients, indicating a common source of infection. Further investigation was conducted to confirm the infection source, identify other potentially exposed patients, and treat any new patients who received an HCV diagnosis. Monitoring frequency of access to drug dispensing systems can help identify staff members with abnormal dispensing patterns, including diversion activities (*1*). U.S. health care facilities are required to prevent, identify, and report any loss, diversion, or theft of controlled substances (*2*).

## Investigation and Results

The first patient, a man in his 60s, was evaluated at the hospital ED for abdominal pain on December 6, 2017, and received injectable narcotic drugs from two nurses. The patient returned to the same ED on January 12, 2018, with history of jaundice and abdominal discomfort. During this visit, the patient had elevated liver enzymes and tested positive for both anti-HCV and HCV RNA. In December 2016, the patient had tested negative for anti-HCV during routine screening for persons born during 1945–1965 and did not have any behavioral risk factors associated with HCV infection acquisition. The two nurses who treated the patient with injectable narcotic drugs had each withdrawn injectable narcotic drugs from the automated drug dispensing system at a frequency that was >3 standard deviations above the mean for all staff members during February 2018. On March 19, 2018, one of the nurses (nurse A) tested positive for anti-HCV using an immunoassay test and tested negative for HCV RNA using a real time reverse transcription–polymerase chain reaction test; a week later, she tested HCV RNA–positive at a level less than the lower limit of detection of 15 IU/mL, too low for viral sequencing. This nurse, who had tested anti-HCV–negative and HCV RNA–negative with a blood donation in 2013, admitted diverting injectable narcotic and antihistamine drugs from patients for personal use during current employment at the hospital ED, though she did not specify the mechanism. On March 27, 2018, the other nurse (nurse B) tested negative for anti-HCV using an immunoassay test. Both nurses tested negative for human immunodeficiency virus (HIV) and hepatitis B virus (HBV) infections.

On December 16, 2017, in the same ED, a woman in her 50s received injectable narcotic drugs for neck pain from nurse A. This patient, who also did not have behavioral risk factors associated with HCV infection acquisition, returned to the same ED on March 23, 2018, with jaundice and tested positive for both anti-HCV and HCV RNA.

CDC’s Division of Viral Hepatitis performed HCV genetic sequencing and phylogenetic analysis on specimens from both ED patients; a high degree of similarity in nucleotide sequences (>96%) between HCV viral variants sampled from two persons indicates a common source of transmission (*3*,*4*). Both patients had HCV genotype 1a that was >96% similar; it was not possible to assess the similarity between the HCV nucleotides in the infected patients and nurse A because HCV RNA titers for nurse A were too low.

Nurse A worked at the ED during August 4, 2017–March 23, 2018. During that period, the hospital identified 2,985 patients who received injectable drugs (i.e., narcotic, sedative, or antihistamine drugs) at the ED while she was on duty, regardless of whether she had been assigned to provide their care. On April 28, 2018, the hospital mailed letters to the 2,762 (93%) living patients who received the injectable drugs when nurse A was on duty, including 208 (7.5%) patients who were treated by nurse A. The letters described potential HCV exposure and offered free testing for HCV, HBV, and HIV infections.

By November 1, 2018, a total of 1,863 (67%) of 2,762 patients had been tested for HCV, HBV, and HIV infections, including 175 (84%) of the 208 patients treated by nurse A. Among those 175 patients, 20 (11%) tested positive for anti-HCV or HCV RNA, including 13 (65%) who had HCV genotype 1a with >96% similarity between their intrahost nucleotide sequences, three (15%) who tested anti-HCV–positive but HCV RNA–negative, and four (20%) who tested HCV RNA–positive with titers below quantification level. Among the remaining 1,688 patients with no record of treatment by nurse A, 65 (4%) tested positive for anti-HCV or HCV RNA, including 49 (75%) with positive anti-HCV and negative HCV RNA, 15 (25%) who had both positive anti-HCV and HCV RNA, which were not genetically related (10 genotype 1a, one genotype 1b, one genotype 2b, and three genotype 3a), and one (1%) with positive RNA titers below quantification level. No screened patients tested positive for HIV, and no new HBV infections were identified. No other health care providers at the ED were offered HCV testing, and no others had provided treatment to a majority of the 13 patients with genetically similar HCV infection.

Twelve of 13 patients with genetically similar HCV RNA specimens had newly diagnosed HCV infection and had received injectable narcotic, sedative, or antihistamine drugs from nurse A during November 22–December 26, 2017 (Figure). One patient was known to have chronic HCV infection and received injectable narcotic drugs from nurse A twice in the ED: first on August 17, 2017, and again on November 8, 2017. It is possible that nurse A acquired the virus from the patient with chronic HCV infection during the November 8 visit and was infectious during November 22–December 26, 2017, during which time at least 12 patients that she treated became infected.

**FIGURE:**
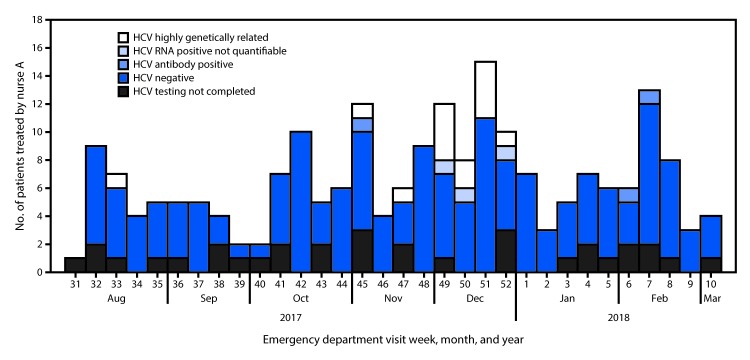
Hepatitis C virus (HCV) infection testing among patients (n = 208*) who were treated by nurse A during their visit to the hospital’s emergency department during August 2017–March 2018 * Three patients had visited and received treatment from nurse A at the emergency department twice. Two of the patients tested negative for both HCV antibodies and HCV RNA; one previously diagnosed chronically HCV-infected patient with genetically similar HCV had received treatment at the emergency department on August 17, 2017, and November 8, 2017.

## Public Health Action

All screened patients with positive HCV RNA results were referred for care, including hepatitis C treatment for those who developed chronic infection. Because of the high risk for HCV infection among patients who received injections from nurse A, the local health department is conducting additional outreach to the remaining 33 (16%) patients who had not been tested for hepatitis C at the time of this analysis. The Washington State Nursing Commission conducted a separate investigation of nurse A’s professional conduct and suspended her practicing license.

## Discussion

An HCV outbreak occurred among patients treated in a Washington ED; transmission likely occurred as the result of unsafe injection practices during drug diversion by a health care provider. Drug diversion by health care providers can pose serious infection risks for patients (*1*). Transmission of HCV from infected health care providers who divert patient drugs has been previously reported, and in some cases those providers have reported unsafe practices including injecting themselves with the patient’s drug, refilling the syringe with water, and injecting water into the patient (*5*,*6*). Some investigations have confirmed transmission of HCV infection from health care providers to patients by identifying genetically similar HCV infections in both the health care providers and infected patients (*3*).

Several epidemiologic findings in this investigation strongly indicate that nurse A was the likely source of infection for the 12 patients with acute HCV infection. First, she had accessed the automated drug dispensing system at a higher frequency than had other staff members and admitted to diverting patient injectable narcotic drugs for personal use. Second, she had seroconverted to anti-HCV–positive after a previous negative test and then tested positive for HCV RNA, indicating recent infection. Finally, having administered injectable narcotic, sedative, or antihistamine drugs to each patient, nurse A was the only common epidemiologic link to 13 patients with genetically similar HCV. The patients with HCV infection who were not cared for by nurse A were infected by strains that were genetically distant from each other and from the HCV 1a strains infecting the group of 13 patients.

Because repeat HCV infection after viral clearance might occur with reexposure to the virus (*7*), it is possible that nurse A could have experienced more than one acute HCV infection between the last negative anti-HCV and HCV RNA tests in 2013 and first positive test in 2018. It is also possible that other patients infected by the nurse were missed by limiting the investigation to the period of this outbreak.

Health care facilities need to develop security measures and to actively monitor drug dispensing systems to detect and prevent narcotic and other drug diversion (*2*,*8*). Protocols to respond to identified drug diversion should address testing of patients at risk for contracting illness and measures to prevent further transmission.

**Summary**

**What is already known about this topic?**

U.S. health care facilities are required to prevent, identify, and report any loss, diversion, or theft of controlled substances. Tampering with injectable narcotic drugs can expose patients to infections.

**What is added by this report?**

Routine surveillance detected acute hepatitis C virus (HCV) infections in two hospital emergency department patients. Investigation identified an outbreak of at least 12 HCV infections in patients who had received opioid injections from a nurse who admitted to diverting injectable narcotic drugs.

**What are the implications for public health practice?**

Health care facilities and public health partners should recognize the potential for infections and other harms from drug diversion and minimize risks by storing controlled substances securely and routinely scrutinizing drug access logs.

## Footnotes

* Drug diversion is the shift of a prescribed substance from the patient for whom it was prescribed to another person for illicit use. https://www.cdc.gov/mmwr/volumes/65/wr/mm6521a4.htm?s_cid=mm6521a4_w.
